# Association of Hemoglobin and Hematocrit Levels during Pregnancy and Maternal Dietary Iron Intake with Allergic Diseases in Children: The Japan Environment and Children’s Study (JECS)

**DOI:** 10.3390/nu13030810

**Published:** 2021-03-01

**Authors:** Limin Yang, Miori Sato, Mayako Saito-Abe, Makoto Irahara, Minaho Nishizato, Hatoko Sasaki, Mizuho Konishi, Kazue Ishitsuka, Hidetoshi Mezawa, Kiwako Yamamoto-Hanada, Kenji Matsumoto, Yukihiro Ohya, on behalf of the Japan Environment and Children’s Study (JECS) Group

**Affiliations:** 1 Allergy Center, National Center for Child Health and Development, Tokyo 157-8535, Japan; sato-m@ncchd.go.jp (M.S.); saito-myk@ncchd.go.jp (M.S.-A.); irahara-m@ncchd.go.jp (M.I.); nishizato-m@ncchd.go.jp (M.N.); sasaki-ht@ncchd.go.jp (H.S.); konishi-m@ncchd.go.jp (M.K.); ishitsuka-k@ncchd.go.jp (K.I.); mezawa-h@ncchd.go.jp (H.M.); yamamoto-k@ncchd.go.jp (K.Y.-H.); matsumoto-k@ncchd.go.jp (K.M.); ohya-y@ncchd.go.jp (Y.O.); 2 Medical Support Center for the Japan Environment and Children’s Study, National Research Institute for Child Health and Development, Tokyo 157-8535, Japan

**Keywords:** hemoglobin, hematocrit, iron intake, allergy, pregnancy

## Abstract

Few epidemiologic studies have examined the role of maternal iron status in allergic diseases in offspring and findings have been inconsistent. We used a large birth cohort in Japan to explore the association of the markers for maternal iron status (maternal hemoglobin, hematocrit and dietary iron intake during pregnancy) with allergy development in offspring during early childhood. We analyzed information on children age 0–3 years from the Japan Environment and Children’s Study (JECS). We used logistic models and generalized estimating equation models to evaluate the effect of maternal hemoglobin and hematocrit levels and dietary iron intake on allergies in children. Models were also fitted with propensity score-matched datasets. Data were collected for a total of 91,247 mother–child pairs. The prevalence (95% confidence interval) of low hemoglobin and hematocrit was 14.0% (13.7–14.2%) and 12.5% (12.3–12.8%), respectively. After adjusting confounders, low hemoglobin and hematocrit during pregnancy were not associated with childhood allergic outcomes. Findings from models with propensity score-matched datasets also indicated that children born to mothers with low hemoglobin or hematocrit levels during pregnancy did not have a higher risk of developing allergic conditions at 3 years old. We found no meaningful associations between low energy adjusted maternal dietary iron intake and allergies in children. In conclusion, using birth cohort data, we found no evidence supporting an association of low maternal hemoglobin, hematocrit and low dietary iron intake with allergy symptoms during early childhood. Further studies with more suitable proxy markers for blood iron status are needed.

## 1. Introduction

Anemia is one of the most common complications in young women, especially during pregnancy. Anemia is considered a risk for many adverse maternal and perinatal outcomes, such as prematurity and low size or birth weight, peripartum blood loss, maternal depression, fetal impairment and maternal and fetal mortality. Anemia can be caused by genetic traits, inadequate food consumption, folate, or vitamin B12 and other diseases such as malaria, schistosomiasis, hookworm infection and HIV infection [1]. The most common cause of anemia is iron deficiency, which accounts for 50% of diagnoses [1]. In 2011, the prevalence of anemia during pregnancy was 38% worldwide [1], of whom about 75% were manifested with iron deficiency [2]. During pregnancy, the plasma volume increases disproportionately to the erythrocyte mass. As a result, pregnant women with a hemoglobin level less than 12 g/dL may experience “physiologic anemia of pregnancy”. It is generally considered that a hemoglobin concentration <11 g/dL in the late first trimester is abnormal [3].

Allergic conditions are common chronic conditions of immunologic dysfunction among children. A recent nationwide survey in Japan indicated that the prevalence among Japanese primary school students of wheeze, allergic rhino-conjunctivitis and eczema was 10.2%, 18.7% and 14.6%, respectively [4]. The prevalence rates have increased over the past decades [5]. Interactions between genetics and environmental exposures affect the development of allergy [6,7].

Iron deficiency anemia in pregnancy has been linked to an inadequate oxygen and nutrition supply to the fetus, which increases the risk of preterm birth and low birth weight [8,9,10,11,12,13]. Evidence suggests that preterm birth and low birth weight can affect the offspring’s lung function and development of allergic diseases in childhood [9,14,15,16,17,18,19,20]. In addition, a recent study demonstrated that low iron concentration interferes with interferon (IFN)-γ receptor signaling, which is a key factor for allergy prevention [21]. Given this situation, low maternal iron status might influence the development of adverse outcomes in children, including allergies. However, to date, few epidemiologic studies have examined the role of maternal iron status on allergic diseases in offspring and the findings have been inconsistent [18,22,23,24].

In this study, we used a large birth cohort in Japan to explore the association of indicator of maternal iron status (maternal hemoglobin, hematocrit concentration and dietary iron intake) during pregnancy with development of allergies in offspring during early childhood.

## 2. Methods

### 2.1. Study Design

Our study population consisted of children between age 0 to 3 years participating in the Japan Environment and Children’s Study (JECS), a nationwide birth cohort study with 104,062 fetal records [25]. The design of the JECS has been described in detail elsewhere [25,26,27]. The JECS was conducted in accordance with the Helsinki Declaration, and approved by the Ministry of the Environment’s Institutional Review Board on Epidemiological Studies (No.100406001, 6 April 2010) and by the Ethics Committees of all participating units and institutions. All participants signed informed consent forms [28,29]. The jecs-ta-201901930-qsn dataset, released by the Program Office in October 2019 was used for this analysis.

### 2.2. Data Collection

**Figure 1:**
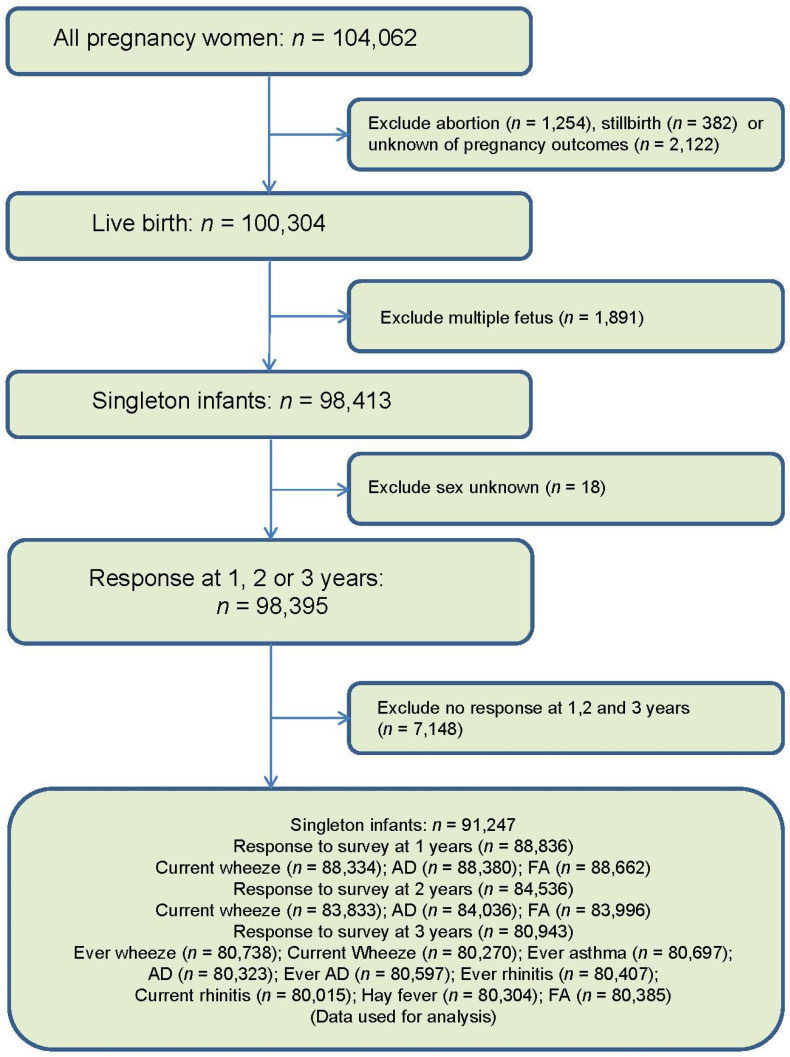
Flow chart of data selection.

The processes of data selection in this study, are shown in Figure 1. Abortion, stillbirth, unknown pregnancy outcomes, twins and triplets were excluded from the analysis. In the selected cohort, we used data from the following questionnaires: (1) Questionnaires completed by women during their first trimester (named MT1 in JECS) and second/third trimester (MT2). These questionnaires were used to collect information regarding maternal medical background, lifestyle and socioeconomic status; (2) questionnaires completed by the parents of children age 1 to 3 years. Hemoglobin and hematocrit were obtained from maternal blood samples collected during the first trimester.

### 2.3. Allergies in Children

Data on allergic outcomes in children were obtained from the questionnaires completed by the caregiver of children age 1 to 3 years. The outcome variables in this study, included wheeze, asthma, atopic dermatitis (AD), rhinitis, hay fever and food allergy. The definitions of allergic outcomes (wheeze, asthma, atopic dermatitis (AD), rhinitis and hay fever), using a modified Japanese-translated version of International Study of Asthma and Allergies in Childhood (ISAAC) [30,31,32], which was validated based on the ISAAC protocol for 6–7 years children with part of modification, are as follows:

- Current wheeze: Caregiver’s report of wheeze in the past 12 months.
- Ever wheeze: Caregiver’s report of wheeze ever.
- Ever asthma: A positive answer to the question “Has your children ever had asthma?”
- Ever rhinitis: Caregiver’s report of sneezing or runny or congested nose ever when the child did not have cold or flu.
- Current rhinitis: Sneezing or a runny or congested nose accompanied by itchy when the child did not have cold or flu in the past 12 months.
- Ever atopic dermatitis: Caregiver’s report of AD ever.
- Current atopic dermatitis: Caregiver’s report of an itchy rash at any time in the past 12 months that occurred at the folds of the elbows, behind the knees, in front of the ankles, under the buttocks, or around the neck, ears, or eyes.
- Hay fever: Caregiver’s report of hay fever ever.
- Food allergy: Caregiver’s report of any allergic symptoms (urticaria, swelling, sneezing, runny nose, itchiness of the mouth, wheezing, cough, vomiting, abdominal pains, fainting, or becoming unconscious within 3 h of food intake).

### 2.4. Hemoglobin, Hematocrit Concentration and Dietary Iron Intake during Pregnancy

As hemoglobin concentrations in our study were obtained during the first trimester, a hemoglobin level less than 11 g/dL was defined as low hemoglobin. A cutoff value of 33% was used for hematocrit level to define low hematocrit.

Dietary Iron intake during pregnancy was obtained from a food frequency questionnaire (FFQ), querying women’s habitual consumption of the listed food items [33]. In the JECS, the FFQ was administered twice during pregnancy, the first time during early pregnancy (FFQ1) and the second time during mid-pregnancy (FFQ2). The FFQ1 queried women’s diet over the past year and the FFQ2 queried the diet during the period of pregnancy. Hence, we used the FFQ2 to evaluate the association of iron intake during pregnancy and allergies in children. Maternal iron intake was adjusted for total energy intake by using the nutrient residuals method [34] and divided into quintile based on the distribution of the residuals. Energy-adjusted Iron intake data from the FFQ1 was used in the model predicting propensity scores (PS) for maternal anemia during pregnancy (described in the statistical analysis section).

### 2.5. Covariates in the Models

Information on the height and weight of pregnant women before pregnancy, maternal age, history of abnormalities in pregnancy, maternal smoking, paternal smoking and maternal history of allergy were obtained from the MT1 questionnaire. Information on parental level of education, family income, pet ownership and maternal drinking were obtained from the MT2 questionnaire. Information on delivery, birth weight, sex and parity were obtained from data according to the medical records transcripts after delivery.

### 2.6. Statistical Analysis

We fitted logistic regression models for allergic outcomes at age 3 years. We performed two steps of adjustment. In the first step, we adjusted for offspring sex only. In the second step, we additionally adjusted for history of abnormalities in pregnancy (experiencing any of the following disorders during previous pregnancy: pregnancy-induced hypertension, pregnancy toxemia and eclampsia, gestational diabetes, placental abruption, ectopic pregnancy, placenta previa, hydatidiform mole and other abnormalities of pregnancy and childbirth), maternal smoking, paternal smoking, maternal history of allergy, body mass index (BMI) before pregnancy, maternal age, maternal level of education, paternal level of education, family income, pet keeping, pregnancy complications(experiencing hypertension, hyperthyroidism, hypothyroidism, diabetes, autoimmune disease, heart disease, kidney disease, hepatitis, cerebral infarction, intracerebral hemorrhage, epilepsy, blood disease, cancer, psychiatric disease, neurological disease, thrombosis or other diseases during current pregnancy), maternal drinking, iron preparations and parity. The confounders adjusted in the models, which are known to be associated with anemia in pregnancy or allergy, were determined according to the published literature [22,23,24] and their availability in the JECS. We checked the collinearity of these confounders with a variance inflation factor value of 5. Model selection was not performed in this study. We assumed that data were missing at random. Missing data for independent variables were imputed using multiple imputation (MI) analysis with a chained equations (MICE) algorithm [35]. Twenty data sets with missing data imputed were generated to obtain pooled odds ratios (ORs). All available confounders in the model fitting allergic outcomes were used in the MI processes. We presented the associations as adjusted ORs and 95% confidence intervals (CI). We do not present any p values for this study owing to the enormous sample size, which can easily lead to very low p values. Given that the outcomes are not completely independent of one another, as well as the exploratory nature of the current study, multiple test adjustment was not performed [36]. Because this study was a secondary analysis using a large cohort, sample size calculation was not performed before analysis.

To explore whether a U-shaped relationship existed, we further developed logistic models with hemoglobin, hematocrit and energy adjusted maternal dietary iron intake treated as continuous variables. Restricted cubic splines with three knots at the 10%, 50% and 90% empirical quantiles were used to relax the linearity assumption for these variables.

We also used a PS approach to obtain a PS-matched dataset. The PS predicting the conditional probability of a participant belonging to the low hemoglobin / hematocrit concentrations group was calculated using logistic models. Variables used in the model to obtain PS included energy adjusted dietary iron intake before pregnancy, maternal age, parity, BMI before pregnancy, history of abnormalities in pregnancy, maternal smoking and maternal drinking. We fit the generalized estimating equation (GEE) models with the matched dataset.

We performed several sensitivity analyses to examine the robustness of our results in the presence of other model adjustments. First, we further adjusted gestational weight gain, as a proxy for energy intake during pregnancy. Second, we added birthweight and gestational age into the multivariable models to account for the potential mediating effect of fetal growth. Third, we assessed whether the effect estimates for allergic outcomes in childhood varied by maternal history of allergy by introducing interaction terms. Finally, for repeated measured outcomes including wheeze, asthma and AD (which were queried at children’s ages 1, 2 and 3 years), we constructed GEE models to evaluate the effect of hemoglobin, hematocrit and energy adjusted maternal dietary iron intake in pregnancy on these outcomes.

All the analyses were performed using R version 3.6.1 software (Institute for Statistics and Mathematics, Vienna, Austria; www.r-project.org; Accessed 01 January 2021). The R packages “MatchIt”, “MICE”, “rms” and “GEE” were used for the PS processes, MI and building GEE models.

## 3. Results

### 3.1. Characteristics of the Study Population

The features studied are summarized in Table S1. Data were collected for a total of 91,247 mother–child pairs. The prevalence of women with low hemoglobin and hematocrit concentrations was 14.0% (95% CI 13.7–14.2%) and 12.5% (12.3–12.8%), respectively. Women were predominantly nonsmokers, younger than age 35 years, had full-term normal delivery, gave birth mainly to infants ≥2500 g and most breastfed their children. The prevalence of current wheeze, AD, rhinitis and food allergy in children age 3 years was 17.4% (17.1–17.6%), 12.3% (12.1–12.5%), 30.0% (29.7–30.3%) and 7.1% (6.9–7.3%), respectively.

Table S2 demonstrates maternal and offspring characteristics according to hemoglobin/hematocrit concentrations during pregnancy. Compared with mothers who had normal hemoglobin/hematocrit concentrations, those with low hemoglobin/hematocrit concentrations were older and more likely to have a history of abnormalities in pregnancy. The presence of low hemoglobin/hematocrit concentrations in pregnancy made cesarean birth more likely. Premature birth, low birth weight, nulliparous and non-breastfeeding were also more common among mothers with low hemoglobin/hematocrit concentrations.

### 3.2. Effect of Low Hemoglobin and Hematocrit in Pregnancy on Allergy in Offspring

The ORs of having allergy at 3 years old are presented in Table 1. The reference group was hemoglobin ≥11 g/dL and hematocrit ≥33%, respectively. After adjusting for confounders, low hemoglobin/hematocrit concentrations during pregnancy were not associated with odds of childhood allergic outcomes in these models. Although some lower limits of the ORs were larger than 1, we do not believe that this indicates a significant association because all ORs were approximately 1; moreover, the large sample size led to extremely narrow 95% CIs. Crude and adjusted ORs did not differ substantially (data not shown). Findings from the models using PS-matched datasets also suggested that children born to mothers with low hemoglobin/hematocrit concentrations during pregnancy did not have a higher risk of developing allergic conditions at 3 years old.

When hemoglobin and hematocrit were treated as continuous variables in the logistic models, no obvious association was observed between hemoglobin or hematocrit concentrations and childhood allergic outcomes (Figures S1 and S2).

In sensitivity analyses, the ORs remained similar when additional adjustment was made for gestational weight gain in multivariable models (data not shown). There were no interactions between hemoglobin/hematocrit concentrations and maternal history of allergic diseases. ORs for women with a history of maternal allergy were similar to those without a maternal allergy history (Table S3). Findings from the GEE models suggested that low hemoglobin or hematocrit in pregnancy was not related to current wheeze, AD or food allergy in early childhood among offspring (Table 2). We did not find a meaningful mediating effect of fetal growth. Inclusion of birth weight and gestational weeks in the models did not materially affect the results (Table S4). The reference group was hemoglobin≥11 g/dL and hematocrit ≥33% in Table 2, Tables S3 and S4.

### 3.3. Role of Maternal Dietary Iron Intake on Allergy in Offspring

After adjusting for confounders, we did not observe meaningful associations between low energy adjusted maternal dietary iron intake and childhood allergies (Table 3 and Figure S3). Further adjusting gestational weight gain (data not shown), birth weight and gestational age (data not shown) and adding an interaction term of maternal allergy history with dietary iron intake (Table S5) did not affect the results. When analysis was limited to those who took iron preparations for anemia during pregnancy (Table S6), children born to women who had a low (or high) energy adjusted maternal dietary iron intake during pregnancy did not have a higher risk of developing allergic conditions at 3 years old. Results from the GEE models also showed that maternal iron intake was not related to the development of wheeze, AD, or food allergy during early childhood (Table 4).

## 4. Discussion

In this study, we used a large birth cohort in Japan to explore the association of the markers for maternal iron status (maternal hemoglobin, hematocrit and dietary iron intake during pregnancy) with allergy development in offspring during early childhood. Our findings suggest that low maternal hemoglobin and hematocrit concentrations in pregnancy were not associated with allergic diseases in offspring during early childhood. In addition, dietary iron intake of mother is unlikely to be related to childhood allergy. 

Although the effects of pregnancy complications on adverse outcomes in offspring have been reported extensively, few studies have examined the influence of iron deficiency anemia in pregnancy on allergies in children. The first report on this topic was from the Avon Longitudinal Study of Parents and Children (ALSPAC), a population-based birth cohort that reported a significant inverse association of prenatal iron status with childhood wheezing and eczema up to 42 months of age, using umbilical cord iron concentrations in over 2000 participants. However, the researchers also indicated that caution should be made when interpreting those results owing to performance of multiple analyses [22]. In this study, we used maternal hemoglobin and hematocrit levels to reflect iron levels in the blood. Our findings are consistent with reports from the United Kingdom (UK) and the Generation R study. One study in the UK investigated the association of maternal iron status with childhood wheeze/atopy and lung function up to age 10 years in a subgroup of children in 157 mother–child pairs from the SEATON (Study of Eczema and Asthma to Observe the influence of Nutrition) birth cohort. In the adjusted model, maternal serum ferritin concentrations at 11 weeks of gestation were not found to be associated with wheeze, hay fever, atopy, atopic eczema and asthma up to 10 years of age. Analysis using hemoglobin concentrations at delivery showed no significant associations with childhood wheeze, asthma or eczema [23]. Similar results were reported in a cohort study from the Netherlands, the Generation R study, a population-based birth cohort. In the models with 6229 mother–child pairs, maternal hemoglobin during pregnancy was not associated with wheezing in early childhood or asthma outcomes at age 6 years [24]. Another analysis in the Generation R study evaluated the associations of maternal hemoglobin and hematocrit levels during pregnancy with lung function measures and asthma among 3672 children. No associations of maternal hemoglobin and hematocrit with current asthma at age 10 years were found [18]. The lack of associations might be explained by the adaptive response of the body. It has been suggested that the placenta might adapt to low hemoglobin and hematocrit concentrations by increasing vascular density, which may maintain normal intrauterine fetal growth, consequently decreasing the risk of developing allergies in early childhood [13].

However, in two studies a significant association of maternal hemoglobin levels with the development of wheeze or asthma in childhood was observed. In a study of 597 mother–child pairs in the United States, maternal anemia was found to be associated with wheeze at 1 and 3 years of age and with asthma at 6 years among mothers with asthma [37]. Another study from Finland analyzed 38,381 cases retrieved from a birth register database. The authors found that mild maternal anemia led to an increased risk of developing asthma in male populations [38]. The differences in results stem from differences in the study populations, children’s ages, blood test times and approaches used in the analysis.

Our large-scale prospective study demonstrated no harmful effects of low iron intake during pregnancy with respect to later risk of childhood allergies in the offspring. This finding was consistent with a report from the SEATON cohort study, which showed that low maternal dietary iron intake (including and excluding iron supplement intake) at 32 weeks of gestation did not increase the odds for developing wheeze, asthma, lung function or atopic outcomes up to 10 years of age [23].

The main strength of this study is the prospective design with large sample size, which allowed us to clarify the temporal association between exposure and outcome. Moreover, the large study population included variables on most of the relevant risk factors involving childhood allergy. Thus, we could control a large number of confounders in the models. In addition, we used a PS approach to balance groups with anemia and normal groups in this observational study, which has been used in epidemiologic studies on childhood allergy.

Some limitations of this study need to be highlighted. First, diagnosis of children’s allergy was based solely on reports of caregiver, which were subject to recall bias. However, the ISAAC questionnaire is a validated questionnaire used extensively in allergy research. Internal consistency of the Japanese version ISAAC questions evaluated by our selected dataset was excellent with an overall Cronbach’s alpha value of 0.88, 0.89 and 0.89 for children at 1, 2 and 3 years. Moreover, the prevalence of allergies observed in the JECS data was consistent with that reported in other studies, lending credibility to the definition of allergies with the ISAAC questions. Second, hemoglobin and hematocrit are not a good biomarker of maternal iron status in pregnancy. Iron deficiency only accounts for 50% of the low hemoglobin concentration in pregnancy. A woman with a low hemoglobin level may not be iron deficient. A recent study evaluating prevalence of iron deficiency in the first trimester among non-anemic pregnancy women found that using standard cutoffs of percent transferrin saturation and/or serum ferritin, 42% of non-anemic women in their first trimester were iron deficient [39]. Plasma ferritin, plasma transferrin saturation, or serum soluble transferrin receptor can better reflect iron status in the blood, as compared with hemoglobin. However, no single marker is considered an ideal proxy to define iron deficiency, owing to limited sensitivity and specificity. Third, we did not adjust paternal allergy status owing to a large amount of missing data for this variable. In addition, we did not evaluate hemoglobin at middle or late pregnancy and childhood iron status owing to a lack of data in the JECS. Studies have also found that children born to mothers with anemia may have normal iron status owing to active placental iron transport [40,41,42]. Finally, true dietary iron intake might not be able to be determined. In the JECS, maternal dietary iron intake was calculated according to the FFQ, which may be less accurate than more detailed methods of dietary assessment, such as weighed food diaries [43].

## 5. Conclusions

We found no evidence supporting an association of low maternal hemoglobin and hematocrit concentration with symptoms of wheeze, asthma, AD, hay fever, allergic rhinitis and food allergy in early childhood. In addition, we did not find any associations between low dietary iron intake during pregnancy and allergic outcomes. The association of maternal hemoglobin and hematocrit levels during pregnancy with lung function in the JECS needs evaluation in the future. Further studies with more suitable proxy markers for blood iron levels are also needed.

## Figures and Tables

**Table 1:** Associations of maternal hemoglobin and hematocrit concentrations with allergic outcomes among children at age 3 years.

	Allergic Outcomes	Model 1 ^†^			Model 2 ^‡^			Model 1 ^†,$^			Model 2 ^‡,$^		
		ORs	95% CI		ORs	95% CI		ORs	95% CI		ORs	95% CI	
			Lower	Upper		Lower	Upper		Lower	Upper		Lower	Upper
Hemoglobin (g/dL)													
<11 vs. ≥11	Ever wheeze	0.98	0.94	1.03	0.97	0.93	1.02	1.03	0.97	1.10	1.04	0.97	1.12
	Current wheeze	1.00	0.94	1.05	1.01	0.95	1.07	1.02	0.94	1.10	1.04	0.96	1.13
	Ever asthma	0.95	0.89	1.02	0.95	0.89	1.03	1.00	0.90	1.11	1.01	0.91	1.13
	AD	0.99	0.93	1.05	0.99	0.93	1.06	0.95	0.87	1.04	0.98	0.89	1.07
	Ever AD	1.01	0.95	1.08	1.02	0.95	1.10	1.03	0.94	1.13	1.07	0.97	1.18
	Ever rhinitis	1.03	0.99	1.08	1.05	1.00	1.10	1.09	1.02	1.16	1.10	1.03	1.18
	Current rhinitis	1.03	0.98	1.08	1.05	1.00	1.10	1.10	1.03	1.18	1.12	1.04	1.20
	Hay fever	1.05	0.94	1.17	1.07	0.96	1.20	1.05	0.90	1.23	1.06	0.90	1.24
	FA	1.01	0.93	1.09	1.02	0.94	1.11	1.01	0.90	1.13	1.03	0.92	1.16
Hematocrit (%)													
<33 vs. ≥33	Ever wheeze	0.96	0.92	1.01	0.96	0.91	1.01	1.01	0.94	1.08	1.02	0.95	1.10
	Current wheeze	0.98	0.92	1.04	1.00	0.94	1.06	1.01	0.92	1.10	1.03	0.95	1.13
	Ever asthma	0.94	0.87	1.01	0.95	0.88	1.03	0.96	0.86	1.07	0.99	0.88	1.11
	AD	0.99	0.92	1.06	0.99	0.92	1.06	0.98	0.89	1.07	1.01	0.92	1.12
	Ever AD	1.01	0.94	1.08	1.03	0.96	1.10	1.04	0.94	1.15	1.08	0.98	1.20
	Ever rhinitis	1.04	0.99	1.09	1.06	1.01	1.11	1.12	1.05	1.20	1.13	1.05	1.21
	Current rhinitis	1.03	0.98	1.08	1.05	1.00	1.10	1.11	1.04	1.19	1.12	1.04	1.20
	Hay fever	1.07	0.95	1.19	1.09	0.97	1.22	1.17	1.00	1.38	1.19	1.01	1.40
	FA	1.02	0.93	1.11	1.02	0.94	1.12	1.01	0.90	1.13	1.02	0.90	1.15

OR: odds ratio; CI: confidence interval; AD: atopic dermatitis; FA: food allergy. ^$^ Generalized estimating equation (GEE) with propensity score matched dataset ^†^ Model 1 adjusted for sex. ^‡^ Model 2 adjusted for sex, history of abnormalities in pregnancy, maternal smoking, paternal smoking, maternal history of allergy, body mass index before pregnancy, maternal age, maternal level of education, paternal level of education, family income, pet keeping, pregnancy complications, maternal drinking, iron preparations and parity.

**Table 2:** Odds ratios from generalized estimating equation models evaluating the association of hemoglobin/hematocrit concentrations in pregnancy with wheeze, atopic dermatitis and food allergy in children.

Allergic Outcomes	Model 1 ^†^			Model 2 ^‡^		
	ORs	95% CI		ORs	95% CI	
		Lower	Upper		Lower	Upper
Hemoglobin (<11 g/dL vs. ≥11 g/dL)						
Current wheeze	1.00	0.97	1.04	0.99	0.95	1.03
AD	1.01	0.97	1.06	1.02	0.97	1.07
FA	0.96	0.91	1.01	0.97	0.92	1.02
Hematocrit (<33% vs. ≥33%)						
Current wheeze	0.98	0.94	1.02	0.98	0.94	1.02
AD	1.02	0.97	1.07	1.02	0.97	1.07
FA	0.97	0.92	1.03	0.97	0.92	1.03

OR: odds ratios; CI: confidence interval; AD: atopic dermatitis; FA: food allergy. ^†^ Model 1 adjusted for sex. ^‡^ Model 2 adjusted for sex, history of abnormalities in pregnancy, maternal smoking, paternal smoking, maternal history of allergy, body mass index before pregnancy, maternal age, maternal level of education, paternal level of education, family income, pet keeping, pregnancy complications, maternal drinking, iron preparations and parity.

**Table 3:** Association of energy adjusted maternal dietary iron intake with allergic outcomes among all selected children at age 3 years.

Allergic Outcomes	Energy Adjusted Maternal Dietary Iron Intake	Model 1 ^†^			Model 2 ^‡^		
		ORs	95% CI		ORs	95% CI	
			Lower	Upper		Lower	Upper
Ever wheeze	Q1	1.00	0.95	1.05	0.99	0.95	1.04
	Q2	0.99	0.95	1.04	0.99	0.94	1.04
	Q3	1.00	-	-	1.00	-	-
	Q4	1.01	0.96	1.06	1.01	0.96	1.06
	Q5	1.00	0.95	1.05	1.02	0.97	1.07
Current wheeze	Q1	0.99	0.94	1.05	0.99	0.94	1.05
	Q2	0.98	0.92	1.04	0.98	0.92	1.04
	Q3	1.00	-	-	1.00	-	-
	Q4	1.01	0.95	1.07	1.01	0.95	1.07
	Q5	1.01	0.95	1.07	1.02	0.96	1.08
Ever asthma	Q1	0.96	0.89	1.04	0.95	0.88	1.03
	Q2	0.96	0.89	1.03	0.95	0.88	1.03
	Q3	1.00	-	-	1.00	-	-
	Q4	0.99	0.92	1.07	1.00	0.93	1.08
	Q5	1.04	0.97	1.12	1.06	0.99	1.14
AD	Q1	0.90	0.85	0.97	0.92	0.86	0.98
	Q2	0.97	0.91	1.03	0.97	0.91	1.04
	Q3	1.00	-	-	1.00	-	-
	Q4	0.97	0.91	1.04	0.97	0.90	1.03
	Q5	0.97	0.91	1.04	0.97	0.91	1.04
Ever AD	Q1	1.03	0.96	1.10	1.03	0.96	1.11
	Q2	1.03	0.96	1.10	1.03	0.96	1.10
	Q3	1.00	-	-	1.00	-	-
	Q4	0.95	0.89	1.02	0.96	0.89	1.02
	Q5	1.00	0.93	1.07	1.01	0.94	1.08
Ever rhinitis	Q1	1.02	0.97	1.07	0.99	0.95	1.04
	Q2	1.02	0.98	1.07	1.01	0.97	1.06
	Q3	1.00	-	-	1.00	-	-
	Q4	1.01	0.96	1.06	1.02	0.97	1.06
	Q5	1.02	0.97	1.07	1.03	0.99	1.08
Current rhinitis	Q1	1.00	0.96	1.05	0.98	0.93	1.03
	Q2	1.01	0.96	1.06	1.00	0.95	1.05
	Q3	1.00	-	-	1.00	-	-
	Q4	1.00	0.95	1.05	1.00	0.96	1.05
	Q5	1.00	0.95	1.05	1.01	0.96	1.06
Hay fever	Q1	1.00	0.89	1.13	0.99	0.88	1.11
	Q2	0.96	0.85	1.08	0.95	0.85	1.07
	Q3	1.00	-	-	1.00	-	-
	Q4	1.04	0.93	1.17	1.04	0.93	1.17
	Q5	1.07	0.95	1.19	1.08	0.96	1.21
FA	Q1	0.99	0.90	1.07	1.00	0.92	1.09
	Q2	0.98	0.90	1.07	0.98	0.90	1.07
	Q3	1.00	-	-	1.00	-	-
	Q4	1.01	0.93	1.10	1.00	0.92	1.09
	Q5	0.99	0.91	1.08	0.98	0.90	1.07

OR: odds ratios; CI: confidence interval; AD: atopic dermatitis; FA: food allergy. ^†^ Model 1 adjusted for sex. ^‡^ Model 2 adjusted for sex, history of abnormalities in pregnancy, maternal smoking, paternal smoking, maternal history of allergy, body mass index before pregnancy, maternal age, maternal level of education, paternal level of education, family income, pet keeping, pregnancy complications, maternal drinking, iron preparations and parity.

**Table 4:** Odds ratios from generalized estimating equation models evaluating the association of energy adjusted maternal dietary iron intake with wheeze, atopic dermatitis and food allergy in children.

Allergic Outcomes	Energy Adjusted Maternal Dietary Iron Intake	Model 1 ^†^			Model 2 ^‡^		
		ORs	95% CI		ORs	95% CI	
			Lower	Upper		Lower	Upper
Current wheeze							
	Q1	0.99	0.95	1.03	0.99	0.95	1.03
	Q2	0.98	0.94	1.01	0.97	0.94	1.01
	Q3	1.00	-	-	1.00	-	-
	Q4	1.02	0.98	1.06	1.03	0.99	1.07
	Q5	1.01	0.97	1.05	1.03	0.99	1.07
AD		1.00	-	-	1.00	-	-
	Q1	0.94	0.90	0.99	0.95	0.91	1.00
	Q2	0.98	0.93	1.03	0.98	0.94	1.03
	Q3	1.00	-	-	1.00	-	-
	Q4	0.98	0.93	1.03	0.98	0.93	1.02
	Q5	1.00	0.95	1.05	0.99	0.95	1.04
FA		1.00	-	-	1.00	-	-
	Q1	0.99	0.94	1.05	1.01	0.96	1.07
	Q2	1.01	0.96	1.07	1.01	0.96	1.07
	Q3	1.00	-	-	1.00	-	-
	Q4	0.98	0.93	1.03	0.97	0.92	1.02
	Q5	0.96	0.91	1.02	0.95	0.90	1.01

OR: odds ratios; CI: confidence interval; AD: atopic dermatitis; FA: food allergy; ^†^ Model 1 adjusted for sex. ^‡^ Model 2 adjusted for sex, history of abnormalities in pregnancy, maternal smoking, paternal smoking, maternal history of allergy, body mass index before pregnancy, maternal age, maternal level of education, paternal level of education, family income, pet keeping, pregnancy complications, maternal drinking, iron preparations and parity.

## Data Availability

Data sharing not applicable.

## Supplementary Materials

The following are available online at https://www.mdpi.com/2072-6643/13/3/810/s1, Figure S1: Summary of generalized linear model for estimating the association of maternal hemoglobin with allergies in children at age 3 years, Figure S2: Summary of generalized linear model for estimating the association of maternal hematocrit with allergies in children at age 3 years, Figure S3: Summary of generalized linear model estimating the association of energy adjusted maternal iron intake with allergies in children at age 3 years, Table S1: Characteristics of variables, Table S2: Characteristics of variables by hemoglobin and hematocrit, Table S3: Generalized linear regression model with an interaction term between maternal hemoglobin or hematocrit concentrations in pregnancy and maternal allergy history evaluating the association of maternal hemoglobin or hematocrit concentrations in pregnancy with allergies in children at age 3 years; Table S4: Models evaluating the association of low hemoglobin or hematocrit concentrations in pregnancy with allergies in children at age 3 years, adding potential mediators, Table S5: Generalized linear regression model with an interaction term between energy adjusted maternal dietary iron intake and maternal allergy history evaluating the association of maternal iron intake with allergies in children at age 3 years, Table S6: Association of energy adjusted maternal dietary iron intake with allergic outcomes at age 3 years among children born to women who took iron preparations during pregnancy.

## Abbreviations

JECS: Japan Environment and Children’s Study; GEE: generalized estimating equation; ISAAC: International Study of Asthma and Allergies in Childhood; FFQ: food frequency questionnaire; MT1: first, First, trimester; MT2: Second/third trimester; MI: multiple imputation; OR: odds ratio; CI: confidence interval; PS: propensity scores; AD: atopic dermatitis.
