# Supplementary material for: Association of Hemoglobin and Hematocrit Levels during Pregnancy and Maternal Dietary Iron Intake with Allergic Diseases in Children: The Japan Environment and Children’s Study (JECS)
Source: Nutrients. 2021 Mar 1;13(3):810. doi: 10.3390/nu13030810 (PMC7999127; doi:10.3390/nu13030810)
Supplement: Supplementary file 1 [file nutrients-13-00810-s001.pdf]

Supplementary material

Figure S1 Summary of generalized linear model for estimating the association of maternal hemoglobin with allergies in children at age 3 years

Hemoglobin was treated as continuous variables. Models adjusted for sex, history of abnormalities in pregnancy, maternal smoking, paternal smoking, maternal history of allergy, body mass index before pregnancy, maternal age, maternal level of education, paternal level of education, family income, pet keeping, pregnancy complications, cesarean birth, obstetric complications, maternal drinking, iron preparations, and parity. Restricted cubic splines with three knots at the 10%, 50%, and 90% empirical quantiles were used to relax the linearity assumption for hemoglobin. Reference for ORs: Hemoglobin =11g/dl. Shaded bands: 95% confidence limits for ORs. ORs: odds ratios.

Figure S2 Summary of generalized linear model for estimating the association of maternal hematocrit with allergies in children at age 3 years

Hematocrit was treated as continuous variables. Models adjusted for sex, history of abnormalities in pregnancy, maternal smoking, paternal smoking, maternal history of allergy, body mass index before pregnancy, maternal age, maternal level of education, paternal level of education, family income, pet keeping, pregnancy complications, maternal drinking, iron preparations, and parity. Restricted cubic splines with three

knots at the 10%, 50%, and 90% empirical quantiles were used to relax the linearity assumption for hematocrit. Reference for ORs: hematocrit=33%. Shaded bands: 95% confidence limits for ORs. ORs: odds ratios.

Figure S3 Summary of generalized linear model estimating the association of energy adjusted maternal iron intake with allergies in children at age 3 years.

Energy adjusted maternal iron intake was treated as a continuous variable. Models adjusted for sex, history of abnormalities in pregnancy, maternal smoking, paternal smoking, maternal history of allergy, body mass index before pregnancy, maternal age, maternal level of education, paternal level of education, family income, pet keeping, pregnancy complications, maternal drinking, iron preparations, and parity. Restricted cubic splines with three knots at the 10%, 50%, and 90% empirical quantiles were used to relax the linearity assumption for energy adjusted maternal iron intake. Reference for ORs: median of energy adjusted iron intake. Shaded bands: 95% confidence limits for ORs. ORs: odds ratios.

Table S1 Characteristics of variables

Table S2 Characteristics of variables by hemoglobin and hematocrit

Table S3 Generalized linear regression model with an interaction term between maternal hemoglobin or hematocrit concentrations in pregnancy and maternal allergy

history evaluating the association of maternal hemoglobin or hematocrit concentrations in pregnancy with allergies in children at age 3 years

Table S4 Models evaluating the association of low hemoglobin or hematocrit concentrations in pregnancy with allergies in children at age 3 years, adding potential mediators

Table S5 Generalized linear regression model with an interaction term between energy adjusted maternal dietary iron intake and maternal allergy history evaluating the association of maternal iron intake with allergies in children at age 3 years

Table S6 Association of energy adjusted maternal dietary iron intake with allergic outcomes at age 3 years among children born to women who took iron preparations during pregnancy

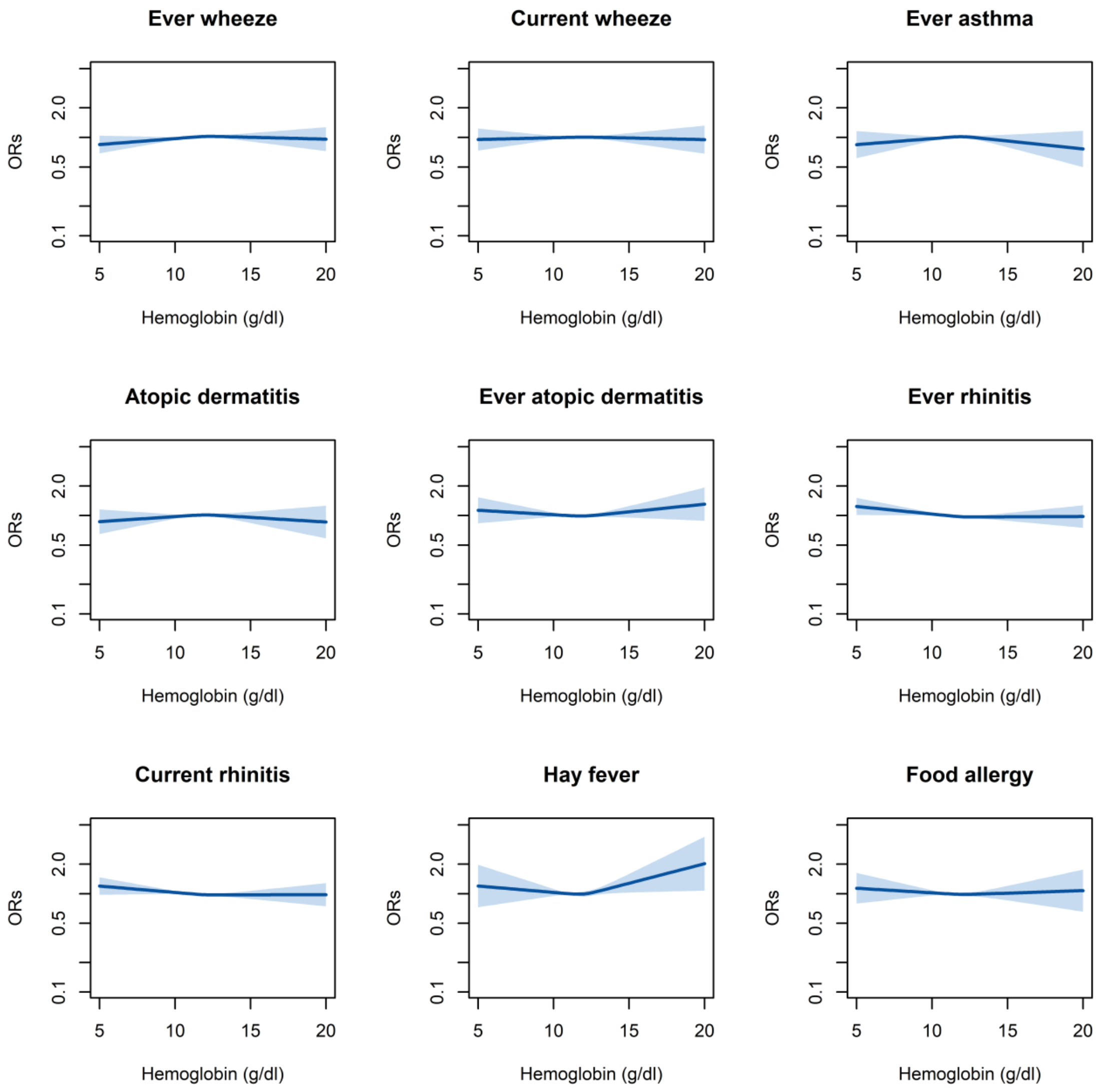
Ever wheeze
Current wheeze
Ever asthma
Atopic dermatitis
Ever atopic dermatitis
Ever rhinitis
Current rhinitis
Hay fever
Food allergy
ORs
0.1
0.5
2.0
5
10
15
20
Hemoglobin (g/dl)

Figure S1

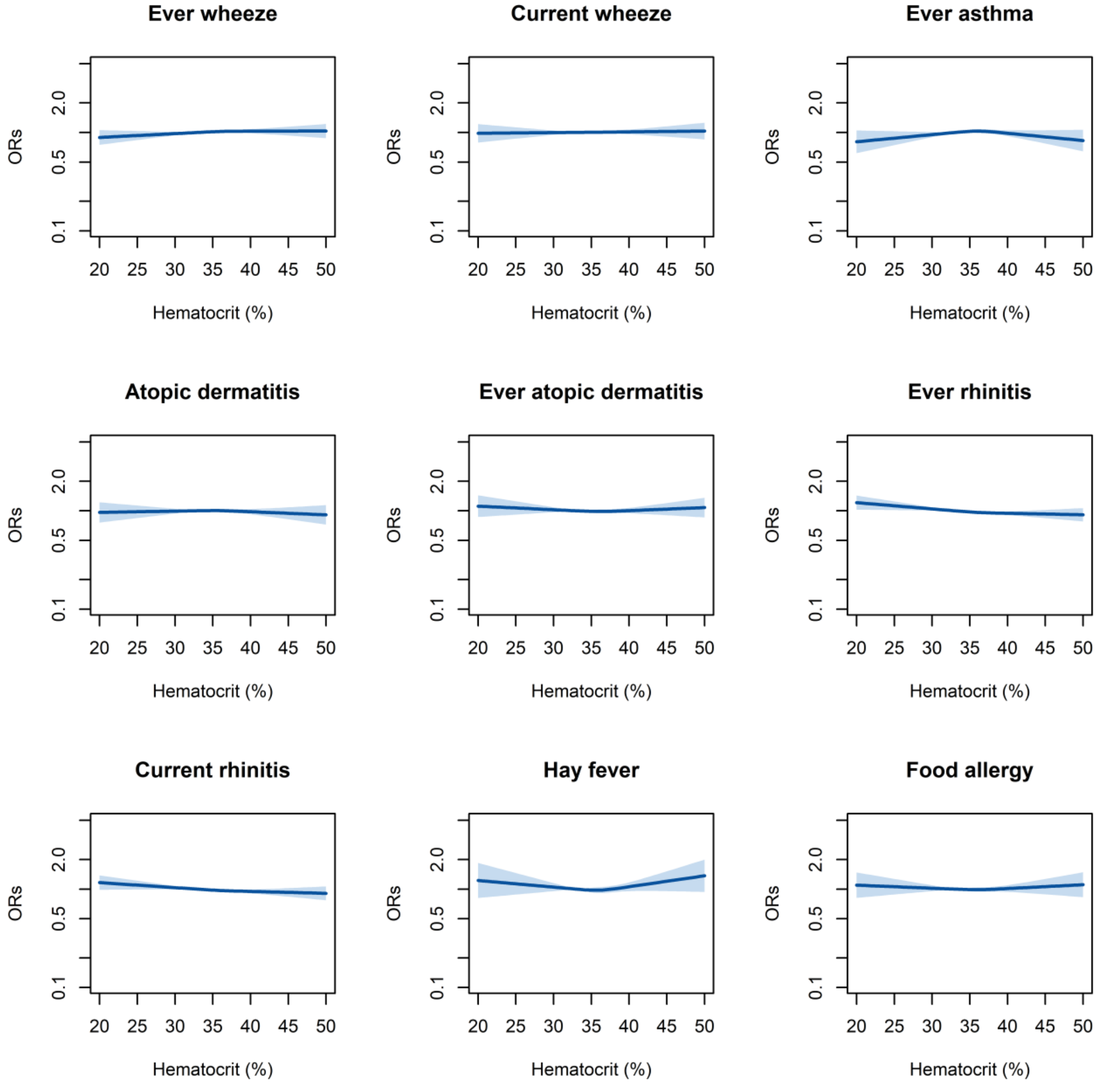

Ever wheeze
Current wheeze
Ever asthma
Atopic dermatitis
Ever atopic dermatitis
Ever rhinitis
Current rhinitis
Hay fever
Food allergy
ORs
0.1
0.5
2.0
20 25 30 35 40 45 50
Hematocrit (%)

Figure S2

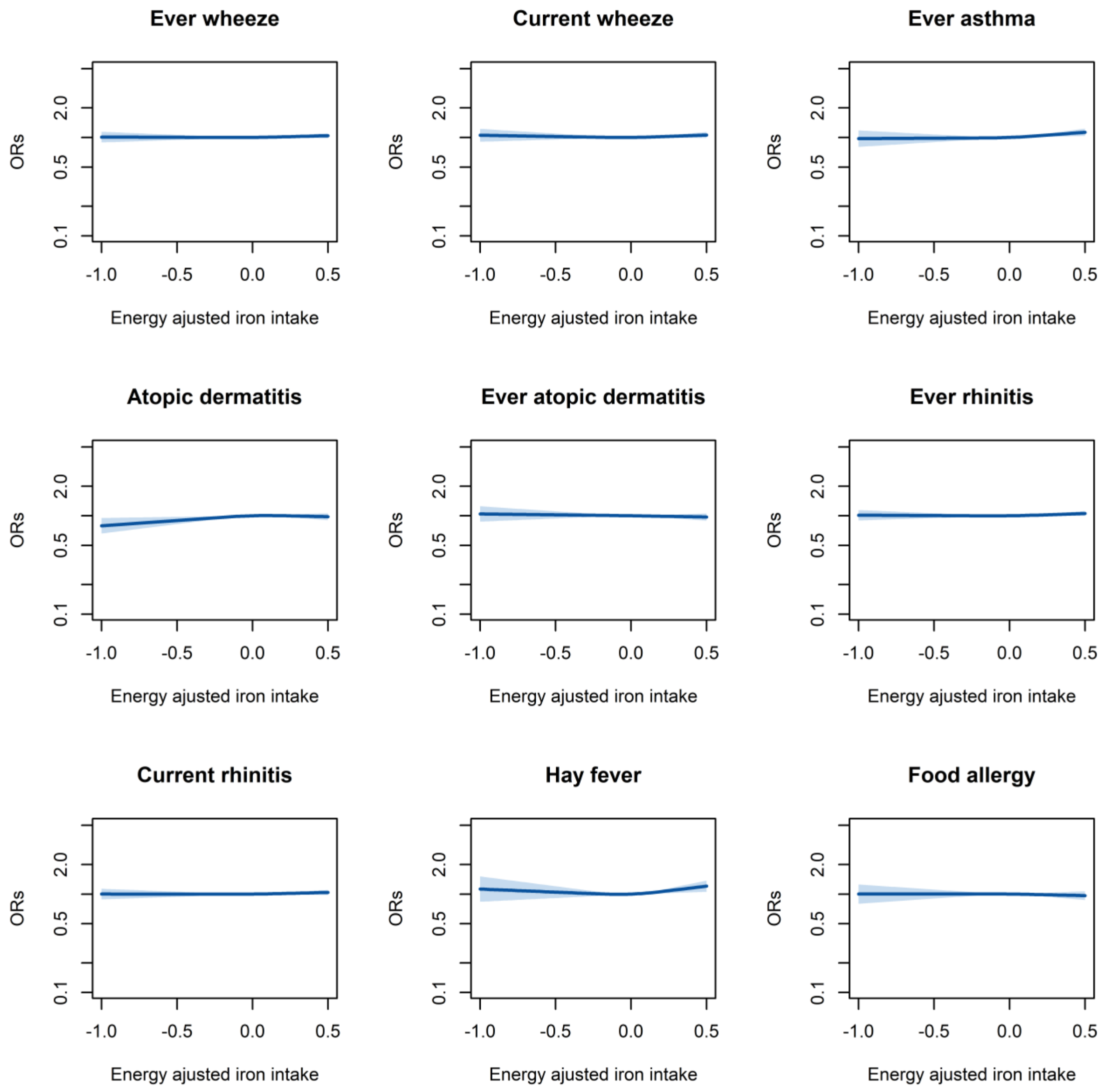
Ever wheeze
Current wheeze
Ever asthma
Atopic dermatitis
Ever atopic dermatitis
Ever rhinitis
Current rhinitis
Hay fever
Food allergy
ORs
0.1
0.5
2.0
-1.0
-0.5
0.0
0.5
Energy ajusted iron intake

Figure S3

Table S1 Characteristics of variables

Variables		n	%	95%CI	
				Lower	Upper
History of abnormality of pregnancy	No	85082	93.9	93.7	94.0
	Yes	5550	6.1	6.0	6.3
	Missing	615			
Maternal smoking	No	86138	95.7	95.6	95.8
	Yes	3864	4.3	4.2	4.4
	Missing	1245			
Paternal smoking	No	47907	53.9	53.6	54.3
	Yes	40920	46.1	45.7	46.4
	Missing	2420			
Maternal history of allergy	No	37949	41.9	41.6	42.2
	Yes	52683	58.1	57.8	58.4
	Missing	615			
Maternal level of education	No	58263	64.7	64.4	65.1
	Yes	31724	35.3	34.9	35.6
	Missing	1260			
Paternal level of education	No	50746	56.7	56.4	57
	Yes	38739	43.3	43.0	43.6
	Missing	1762			
Family income (million yen/per year)	>=4	50869	60.4	60.0	60.7
	<4	33415	39.6	39.3	40.0
	Missing	6963			
Pet keeping	No	69569	77.2	77.0	77.5
	Yes	20489	22.8	22.5	23.0
	Missing	1189			
Pregnancy complications	No	75899	84.9	84.7	85.2
	Yes	13450	15.1	14.8	15.3
	Missing	1898			
Birth weight	>=2500g	83767	92.1	91.9	92.2
	<2500g	7219	7.9	7.8	8.1
	Missing	261			
Sex	Boys	46824	51.3	51.0	51.6
	Girls	44423	48.7	48.4	49.0
	Missing	0			
Gestational age (weeks)	>=37	86886	95.4	95.3	95.6
	<37	4156	4.6	4.4	4.7
	Missing	205			
BMI before pregnancy	<25	81701	89.6	89.4	89.8
	>=25	9479	10.4	10.2	10.6
	Missing	67			
Maternal age (years)	<35	68304	75.4	75.1	75.7
	>=35	22318	24.6	24.3	24.9
	Missing	625			
Maternal drinking	No	87256	97.2	97.1	97.3
	Yes	2505	2.8	2.7	2.9
	Missing	1486			
Iron preparations	No	80229	87.9	87.7	88.1
	Yes	11018	12.1	11.9	12.3
	Missing	0			
Parity	Nulliparous	36438	40.9	40.6	41.2

	Multipara	52620	59.1	58.8	59.4
	Missing	2189			
Ever wheeze (3y)	No	56181	69.6	69.3	69.9
	Yes	24557	30.4	30.1	30.7
	Missing	10509			
Current wheeze (3y)	No	66336	82.6	82.4	82.9
	Yes	13934	17.4	17.1	17.6
	Missing	10977			
Ever asthma (3y)	No	72755	90.2	90.0	90.4
	Yes	7942	9.8	9.6	10.0
	Missing	10550			
AD (3y)	No	70442	87.7	87.5	87.9
	Yes	9881	12.3	12.1	12.5
	Missing	10924			
Ever AD (3y)	No	71558	88.8	88.6	89.0
	Yes	9039	11.2	11.0	11.4
	Missing	10650			
Ever rhinitis (3y)	No	53399	66.4	66.1	66.7
	Yes	27008	33.6	33.3	33.9
	Missing	10840			
Current rhinitis (3y)	No	56000	70.0	69.7	70.3
	Yes	24015	30.0	29.7	30.3
	Missing	11232			
Hay fever (3y)	No	77275	96.2	96.1	96.4
	Yes	3029	3.8	3.6	3.9
	Missing	10943			
FA (3y)	No	74668	92.9	92.7	93.1
	Yes	5717	7.1	6.9	7.3
	Missing	10862			
Current wheeze (2y)	No	63703	76.0	75.7	76.3
	Yes	20130	24.0	23.7	24.3
	Missing	7414			
AD (2y)	No	73043	86.9	86.7	87.1
	Yes	10993	13.1	12.9	13.3
	Missing	7211			
FA (2y)	No	76262	90.8	90.6	91.0
	Yes	7734	9.2	9.0	9.4
	Missing	7251			
Current wheeze (2y)	No	71030	80.4	80.1	80.7
	Yes	17304	19.6	19.3	19.9
	Missing	2913			
AD (1y)	No	76612	86.7	86.5	86.9
	Yes	11768	13.3	13.1	13.5
	Missing	2867			
FA (1y)	No	74932	84.5	84.3	84.8
	Yes	13730	15.5	15.2	15.7
	Missing	2585			
Iron intake before pregnancy (mg)	Median (IQR)	6.5 (5.0-8.4)		Missing: 638	
Iron intake during pregnancy (mg)	Median (IQR)	6.1 (4.7-7.8)		Missing: 1166	
Iron intake by stratified energy adjusted iron intake	n	Mean (SD)			

Q1	17922	5.0 (2.7)			
Q2	18081	5.7 (2.2)			
Q3	18126	6.4 (2.7)			
Q4	18192	7.2 (2.8)			
Q5	18136	8.9 (5.1)			
Hemoglobin	No	70200	86.0	85.8	86.3
	Yes	11404	14.0	13.7	14.2
	Missing	9643			
	Median (IQR)	12.0 (11.4-12.6)			
Hematocrit	No	71376	87.5	87.2	87.7
	Yes	10228	12.5	12.3	12.8
	Missing	9643			
	Median (IQR)	36.0 (34.2-37.8)			

AD: atopic dermatitis; FA: food allergy; BMI, body mass index; IQR: interquartile range.

Table S2 Characteristics of variables by hemoglobin and hematocrit

Variables		Hemoglobin								Hematocrit							
		≥11 g/dl				<11 g/dl				≥33%				<33%			
		n	%	95%CI		n	%	95%CI		n	%	95%CI		n	%	95%CI	
				Lower	Upper			Lower	Upper			Lower	Upper			Lower	Upper
History of abnormality	No	65590	93.8	93.6	94.0	10694	94.1	93.7	94.6	66667	93.8	93.6	94.0	9617	94.4	93.9	94.8
of pregnancy	Yes	4317	6.2	6.0	6.4	667	5.9	5.4	6.3	4415	6.2	6.0	6.4	569	5.6	5.2	6.1
Maternal smoking	No	66553	95.8	95.7	96.0	10816	95.8	95.4	96.1	67657	95.8	95.7	96.0	9712	95.9	95.5	96.3
	Yes	2888	4.2	4.0	4.3	476	4.2	3.9	4.6	2949	4.2	4.0	4.3	415	4.1	3.7	4.5
Paternal smoking	No	37249	54.3	53.9	54.7	5947	53.3	52.4	54.2	37820	54.2	53.9	54.6	5376	53.7	52.7	54.7
	Yes	31349	45.7	45.3	46.1	5211	46.7	45.8	47.6	31930	45.8	45.4	46.1	4630	46.3	45.3	47.3
Maternal history of allergy	No	29160	41.7	41.3	42.1	4817	42.4	41.5	43.3	29662	41.7	41.4	42.1	4315	42.4	41.4	43.3
	Yes	40747	58.3	57.9	58.7	6544	57.6	56.7	58.5	41420	58.3	57.9	58.6	5871	57.6	56.7	58.6
Maternal level of education	High	45309	65.4	65.0	65.7	7106	63.2	62.3	64.1	45911	65.1	64.8	65.5	6504	64.4	63.5	65.3
	Low	24012	34.6	34.3	35.0	4143	36.8	35.9	37.7	24562	34.9	34.5	35.2	3593	35.6	34.7	36.5
Paternal level of education	High	39529	57.3	56.9	57.7	6152	55.0	54.1	56.0	40046	57.1	56.7	57.5	5635	56.1	55.2	57.1
	Low	29468	42.7	42.3	43.1	5028	45.0	44.0	45.9	30094	42.9	42.5	43.3	4402	43.9	42.9	44.8
Family income	>=4	39550	60.7	60.3	61.1	6300	60.2	59.2	61.1	40110	60.6	60.2	60.9	5740	60.9	59.9	61.9
(million yen/per year)	<4	25629	39.3	38.9	39.7	4168	39.8	38.9	40.8	26116	39.4	39.1	39.8	3681	39.1	38.1	40.1
Pet keeping	No	53704	77.4	77.1	77.7	8798	78.1	77.3	78.9	54590	77.4	77.1	77.7	7912	78.3	77.5	79.1
	Yes	15678	22.6	22.3	22.9	2465	21.9	21.1	22.7	15949	22.6	22.3	22.9	2194	21.7	20.9	22.5
Pregnancy complications	No	58358	84.9	84.6	85.2	9506	84.8	84.1	85.4	59293	84.8	84.5	85.1	8571	85.3	84.6	86.0
	Yes	10389	15.1	14.8	15.4	1705	15.2	14.6	15.9	10617	15.2	14.9	15.5	1477	14.7	14.0	15.4
Birth weight (g)	>=2500	64443	92.1	91.8	92.3	10502	92.4	91.9	92.9	65499	92.0	91.8	92.2	9446	92.7	92.1	93.2
	<2500	5564	7.9	7.7	8.2	865	7.6	7.1	8.1	5681	8.0	7.8	8.2	748	7.3	6.8	7.9
Sex	Boys	36202	51.6	51.2	51.9	5629	49.4	48.4	50.3	36831	51.6	51.2	52.0	5000	48.9	47.9	49.9
	Girls	33998	48.4	48.1	48.8	5775	50.6	49.7	51.6	34545	48.4	48.0	48.8	5228	51.1	50.1	52.1
gestational age (weeks)	>=37	66862	95.4	95.3	95.6	10843	95.3	94.9	95.7	67933	95.4	95.2	95.5	9772	95.8	95.4	96.1
	<37	3189	4.6	4.4	4.7	534	4.7	4.3	5.1	3291	4.6	4.5	4.8	432	4.2	3.9	4.6
BMI before pregnancy	>=25	62268	88.8	88.5	89.0	10821	94.9	94.5	95.3	63275	88.7	88.5	88.9	9814	96.0	95.6	96.3
	<25	7890	11.2	11.0	11.5	582	5.1	4.7	5.5	8059	11.3	11.1	11.5	413	4.0	3.7	4.4
Maternal age (years)	<35	53246	76.2	75.9	76.5	8022	70.6	69.8	71.5	54026	76.0	75.7	76.3	7242	71.1	70.2	72.0
	>=35	16654	23.8	23.5	24.1	3336	29.4	28.5	30.2	17050	24.0	23.7	24.3	2940	28.9	28.0	29.8

Maternal drinking	No	67350	97.4	97.3	97.5	10860	96.8	96.4	97.1	68464	97.4	97.3	97.5	9746	96.7	96.3	97.0
	Yes	1815	2.6	2.5	2.7	361	3.2	2.9	3.6	1841	2.6	2.5	2.7	335	3.3	3.0	3.7
Iron preparations	No	64071	91.3	91.1	91.5	7880	69.1	68.2	69.9	64737	90.7	90.5	90.9	7214	70.5	69.6	71.4
	Yes	6129	8.7	8.5	8.9	3524	30.9	30.1	31.8	6639	9.3	9.1	9.5	3014	29.5	28.6	30.4
Parity	Nulliparous	27991	40.9	40.5	41.2	4084	36.6	35.7	37.5	28208	40.5	40.1	40.9	3867	38.6	37.7	39.6
	Multipara	40491	59.1	58.8	59.5	7089	63.4	62.5	64.3	41439	59.5	59.1	59.9	6141	61.4	60.4	62.3
Ever wheeze (3y)	No	43234	69.4	69.0	69.8	7068	70.0	69.1	70.9	43934	69.3	69.0	69.7	6368	70.4	69.5	71.4
	Yes	19062	30.6	30.2	31.0	3032	30.0	29.1	30.9	19419	30.7	30.3	31.0	2675	29.6	28.6	30.5
Current wheeze (3y)	No	51148	82.6	82.3	82.9	8307	82.7	82.0	83.5	51997	82.5	82.2	82.8	7458	83.0	82.2	83.7
	Yes	10795	17.4	17.1	17.7	1732	17.3	16.5	18.0	10996	17.5	17.2	17.8	1531	17.0	16.3	17.8
Ever asthma (3y)	No	56084	90.1	89.8	90.3	9151	90.6	90.1	91.2	57033	90.1	89.8	90.3	8202	90.8	90.2	91.4
	Yes	6181	9.9	9.7	10.2	944	9.4	8.8	9.9	6292	9.9	9.7	10.2	833	9.2	8.6	9.8
AD (3y)	No	54321	87.6	87.4	87.9	8818	87.8	87.2	88.5	55233	87.6	87.4	87.9	7906	87.9	87.2	88.5
	Yes	7661	12.4	12.1	12.6	1223	12.2	11.5	12.8	7792	12.4	12.1	12.6	1092	12.1	11.5	12.8
Ever AD (3y)	No	55224	88.8	88.5	89.0	8939	88.7	88.1	89.3	56163	88.8	88.5	89.0	8000	88.7	88.1	89.4
	Yes	6971	11.2	11.0	11.5	1136	11.3	10.7	11.9	7092	11.2	11.0	11.5	1015	11.3	10.6	11.9
Ever rhinitis (3y)	No	41336	66.6	66.2	67.0	6628	65.9	65.0	66.8	42055	66.6	66.3	67.0	5909	65.7	64.7	66.7
	Yes	20709	33.4	33.0	33.8	3429	34.1	33.2	35.0	21056	33.4	33.0	33.7	3082	34.3	33.3	35.3
Current rhinitis (3y)	No	43296	70.1	69.8	70.5	6963	69.6	68.7	70.5	44038	70.1	69.8	70.5	6221	69.6	68.6	70.5
	Yes	18450	29.9	29.5	30.2	3044	30.4	29.5	31.3	18771	29.9	29.5	30.2	2723	30.4	29.5	31.4
Hay fever (3y)	No	59653	96.3	96.1	96.4	9651	96.1	95.7	96.5	60669	96.3	96.1	96.4	8635	96.1	95.6	96.4
	Yes	2302	3.7	3.6	3.9	393	3.9	3.5	4.3	2340	3.7	3.6	3.9	355	3.9	3.6	4.4
FA (3y)	No	57605	92.8	92.6	93.0	9329	92.9	92.3	93.4	58592	92.9	92.7	93.1	8342	92.8	92.2	93.3
	Yes	4438	7.2	7.0	7.4	717	7.1	6.6	7.7	4508	7.1	6.9	7.3	647	7.2	6.7	7.8
Current wheeze (2y)	No	48968	75.8	75.5	76.2	8027	76.6	75.7	77.4	49776	75.8	75.5	76.1	7219	76.8	76.0	77.7
	Yes	15592	24.2	23.8	24.5	2456	23.4	22.6	24.3	15873	24.2	23.9	24.5	2175	23.2	22.3	24.0
AD (2y)	No	56248	86.9	86.7	87.2	9103	86.6	85.9	87.2	57173	86.9	86.6	87.2	8178	86.7	86.0	87.4
	Yes	8460	13.1	12.8	13.3	1414	13.4	12.8	14.1	8618	13.1	12.8	13.4	1256	13.3	12.6	14.0
FA (2y)	No	58674	90.7	90.5	90.9	9562	90.9	90.4	91.5	59668	90.7	90.5	90.9	8568	90.9	90.3	91.5
	Yes	6009	9.3	9.1	9.5	952	9.1	8.5	9.6	6105	9.3	9.1	9.5	856	9.1	8.5	9.7
Current wheeze (2y)	No	54677	80.4	80.1	80.7	8822	80.0	79.2	80.7	55538	80.3	80.0	80.6	7961	80.4	79.6	81.1
	Yes	13319	19.6	19.3	19.9	2212	20.0	19.3	20.8	13585	19.7	19.4	20.0	1946	19.6	18.9	20.4
AD (1y)	No	59025	86.8	86.5	87.0	9556	86.6	85.9	87.2	60019	86.8	86.5	87.0	8562	86.4	85.7	87.1
	Yes	9008	13.2	13.0	13.5	1479	13.4	12.8	14.1	9138	13.2	13.0	13.5	1349	13.6	12.9	14.3

FA (1y)	No	57554	84.3	84.1	84.6	9439	85.2	84.6	85.9	58541	84.4	84.1	84.7	8452	85.0	84.3	85.7
	Yes	10679	15.7	15.4	15.9	1634	14.8	14.1	15.4	10825	15.6	15.3	15.9	1488	15.0	14.3	15.7

AD: atopic dermatitis; FA: food allergy; BMI, body mass index.

Table S3 Generalized linear regression model with an interaction term between maternal hemoglobin or hematocrit concentrations in pregnancy and maternal allergy history evaluating the association of maternal hemoglobin or hematocrit concentrations in pregnancy with allergies in children at age 3 years

Allergic outcomes	Maternal allergy history	Maternal anemia[#]					
		Hemoglobin <11 g/dl vs. ≥11 g/dl			Hematocrit <33% vs. ≥33%		
			95%CI			95%CI	
		ORs	Lower	Upper	ORs	Lower	Upper
Ever wheeze	No	1.00	0.93	1.08	0.96	0.89	1.04
	Yes	0.96	0.90	1.02	0.97	0.90	1.03
Current wheeze	No	1.01	0.92	1.11	0.97	0.88	1.07
	Yes	1.01	0.94	1.09	1.02	0.94	1.10
Ever asthma	No	0.97	0.86	1.09	0.94	0.83	1.07
	Yes	0.93	0.85	1.02	0.94	0.86	1.04
AD	No	0.92	0.82	1.04	0.90	0.79	1.02
	Yes	1.00	0.92	1.08	1.01	0.93	1.09
Ever AD	No	0.96	0.85	1.08	0.99	0.87	1.12
	Yes	1.04	0.96	1.14	1.03	0.95	1.13
Ever rhinitis	No	1.09	1.02	1.17	1.09	1.01	1.17
	Yes	1.03	0.97	1.09	1.04	0.98	1.11
Current rhinitis	No	1.08	1.01	1.17	1.07	0.99	1.16
	Yes	1.03	0.97	1.09	1.03	0.97	1.10
Hay fever	No	1.00	0.81	1.24	1.06	0.85	1.31
	Yes	1.08	0.95	1.23	1.08	0.94	1.24
FA	No	0.97	0.84	1.13	0.99	0.86	1.15
	Yes	1.03	0.93	1.13	1.02	0.92	1.13

OR: odds ratios; CI: confidence interval; AD: atopic dermatitis; FA: food allergy.

[#]Models adjusted for sex, history of abnormalities in pregnancy, maternal smoking, paternal smoking, body mass index before pregnancy, maternal age, maternal level of education, paternal level of education, family income, pet keeping, pregnancy complications, maternal drinking, iron preparations, parity, and an interaction term between maternal anemia and maternal allergy history.

Table S4 Models evaluating the association of low hemoglobin or hematocrit concentrations in pregnancy with allergies in children at age 3 years, adding potential mediators

Allergic outcomes	Hemoglobin# <11 g/dl vs. ≥11 g/dl			Hematocrit# <33% vs. ≥33%		
	ORs	95%CI		ORs	95%CI	
		Lower	Upper		Lower	Upper
Ever wheeze	0.97	0.93	1.02	0.96	0.91	1.01
Current wheeze	1.01	0.95	1.07	1.00	0.94	1.06
Ever asthma	0.95	0.89	1.03	0.95	0.88	1.03
AD	0.99	0.93	1.06	0.99	0.92	1.06
Ever AD	1.02	0.95	1.10	1.03	0.96	1.10
Ever rhinitis	1.05	1.00	1.10	1.06	1.01	1.11
Current rhinitis	1.05	1.00	1.10	1.05	1.00	1.10
Hay fever	1.07	0.96	1.20	1.09	0.97	1.22
FA	1.02	0.94	1.11	1.02	0.93	1.12

OR: odds ratios; CI: confidence interval; AD: atopic dermatitis; FA: food allergy.

#Model adjusted for gestational age, birth weight, sex, history of abnormalities in pregnancy, maternal smoking, paternal smoking, maternal history of allergy, body mass index before pregnancy, maternal age, maternal level of education, paternal level of education, family income, pet keeping, pregnancy complications, maternal drinking, iron preparations, and parity,

Table S5 Generalized linear regression model with an interaction term between energy adjusted maternal dietary iron intake and maternal allergy history evaluating the association of maternal iron intake with allergies in children at age 3 years

Allergic outcomes	Energy adjusted maternal dietary iron intake	Maternal allergy history (-)			Maternal allergy history (+)		
		ORs	95%CI		ORs	95%CI	
			Lower	Upper		Lower	Upper
Ever wheeze	Q1	1.00	0.92	1.08	0.98	0.92	1.04
	Q2	0.97	0.90	1.05	0.99	0.93	1.05
	Q3	1.00	-	-	1.00	-	-
	Q4	1.00	0.93	1.08	0.96	0.90	1.02
	Q5	1.00	0.93	1.08	1.01	0.95	1.08
Current wheeze	Q1	1.02	0.93	1.12	0.97	0.90	1.04
	Q2	0.97	0.88	1.07	0.99	0.92	1.06
	Q3	1.00	-	-	1.00	-	-
	Q4	0.98	0.89	1.09	0.95	0.89	1.03
	Q5	1.02	0.93	1.13	1.01	0.94	1.08
Ever asthma	Q1	1.03	0.91	1.17	0.98	0.90	1.08
	Q2	0.96	0.85	1.09	0.94	0.86	1.03
	Q3	1.00	-	-	1.00	-	-
	Q4	0.97	0.85	1.10	0.94	0.86	1.03
	Q5	1.09	0.96	1.23	1.05	0.96	1.15
AD	Q1	0.98	0.88	1.10	1.07	0.98	1.16
	Q2	0.86	0.76	0.97	1.00	0.92	1.09
	Q3	1.00	-	-	1.00	-	-
	Q4	0.99	0.88	1.11	1.01	0.93	1.10
	Q5	0.96	0.86	1.08	1.04	0.95	1.12
Ever AD	Q1	1.00	0.89	1.13	1.07	0.99	1.17
	Q2	1.04	0.92	1.18	1.10	1.01	1.19
	Q3	1.00	-	-	1.00	-	-
	Q4	1.03	0.91	1.16	1.10	1.01	1.20
	Q5	1.03	0.91	1.16	1.07	0.98	1.17
Ever rhinitis	Q1	1.04	0.96	1.12	0.95	0.90	1.01
	Q2	0.98	0.91	1.06	0.98	0.92	1.04
	Q3	1.00	-	-	1.00	-	-
	Q4	1.01	0.94	1.09	0.99	0.93	1.05
	Q5	1.00	0.93	1.08	1.03	0.97	1.09
Current rhinitis	Q1	1.05	0.98	1.14	0.96	0.90	1.02
	Q2	0.98	0.91	1.06	0.97	0.91	1.03
	Q3	1.00	-	-	1.00	-	-
	Q4	1.01	0.93	1.09	0.98	0.93	1.05
	Q5	1.00	0.92	1.08	1.01	0.95	1.08
Hay fever	Q1	0.91	0.73	1.14	0.98	0.85	1.12
	Q2	0.92	0.74	1.15	0.95	0.83	1.09
	Q3	1.00	-	-	1.00	-	-
	Q4	0.91	0.72	1.13	0.92	0.80	1.05
	Q5	0.99	0.80	1.24	1.05	0.92	1.19
FA	Q1	1.08	0.92	1.26	0.97	0.87	1.07
	Q2	1.11	0.95	1.30	0.96	0.86	1.06
	Q3	1.00	-	-	1.00	-	-
	Q4	1.09	0.94	1.28	0.94	0.85	1.04
	Q5	1.07	0.92	1.25	0.94	0.85	1.04

OR: odds ratios; CI: confidence interval; AD: atopic dermatitis; FA: food allergy.

[#]Model adjusted for sex, history of abnormalities in pregnancy, maternal smoking, paternal smoking, body mass index before pregnancy, maternal age, maternal level of education, paternal level of education, family income, pet keeping, pregnancy complications, maternal drinking, iron preparations, parity, and an interaction term between maternal iron intake and maternal allergy history.

Table S6 Association of energy adjusted maternal dietary iron intake with allergic outcomes at age 3 years among children born to women who took iron preparations during pregnancy

Allergic outcomes	Energy adjusted maternal dietary iron intake	Model 1†			Model 2‡		
		ORs	95%CI		ORs	95%CI	
			Lower	Upper		Lower	Upper
Ever wheeze	Q1	0.94	0.82	1.07	0.94	0.82	1.07
	Q2	0.94	0.83	1.08	0.94	0.82	1.07
	Q3	1.00	-	-	1.00	-	-
	Q4	0.98	0.86	1.12	1.00	0.88	1.14
	Q5	0.85	0.74	0.97	0.87	0.76	0.99
Current wheeze	Q1	0.93	0.79	1.10	0.93	0.79	1.10
	Q2	0.92	0.79	1.09	0.92	0.78	1.08
	Q3	1.00	-	-	1.00	-	-
	Q4	1.01	0.86	1.19	1.03	0.87	1.20
	Q5	0.87	0.74	1.03	0.88	0.75	1.04
Ever asthma	Q1	0.86	0.70	1.06	0.84	0.69	1.04
	Q2	0.91	0.74	1.11	0.90	0.74	1.10
	Q3	1.00	-	-	1.00	-	-
	Q4	0.89	0.72	1.08	0.90	0.74	1.11
	Q5	0.83	0.67	1.02	0.86	0.70	1.06
AD	Q1	1.03	0.85	1.24	1.05	0.87	1.27
	Q2	1.02	0.85	1.23	1.03	0.85	1.24
	Q3	1.00	-	-	1.00	-	-
	Q4	0.97	0.80	1.17	0.97	0.80	1.17
	Q5	1.00	0.83	1.20	1.01	0.84	1.22
Ever AD	Q1	1.08	0.89	1.31	1.10	0.90	1.34
	Q2	1.02	0.84	1.25	1.02	0.84	1.25
	Q3	1.00	-	-	1.00	-	-
	Q4	0.89	0.73	1.09	0.90	0.73	1.10
	Q5	0.99	0.81	1.21	1.01	0.83	1.24
Ever rhinitis	Q1	1.02	0.90	1.16	1.00	0.88	1.14
	Q2	1.03	0.91	1.18	1.02	0.90	1.16
	Q3	1.00	-	-	1.00	-	-
	Q4	0.98	0.86	1.12	0.99	0.87	1.13
	Q5	1.03	0.90	1.17	1.04	0.92	1.19
Current rhinitis	Q1	1.00	0.88	1.15	0.98	0.86	1.13
	Q2	0.97	0.84	1.10	0.96	0.83	1.09
	Q3	1.00	-	-	1.00	-	-
	Q4	0.96	0.84	1.09	0.97	0.85	1.11
	Q5	1.04	0.91	1.19	1.05	0.92	1.21
Hay fever	Q1	0.85	0.61	1.20	0.84	0.60	1.18
	Q2	1.00	0.72	1.38	0.98	0.71	1.36
	Q3	1.00	-	-	1.00	-	-
	Q4	0.89	0.64	1.24	0.90	0.65	1.25
	Q5	1.10	0.80	1.51	1.14	0.83	1.57
FA	Q1	1.28	1.01	1.62	1.32	1.04	1.67
	Q2	1.21	0.96	1.53	1.22	0.96	1.54
	Q3	1.00	-	-	1.00	-	-
	Q4	1.02	0.80	1.30	1.02	0.80	1.30
	Q5	1.10	0.86	1.40	1.08	0.85	1.38

OR: odds ratios; CI: confidence interval; AD: atopic dermatitis; FA: food allergy.
†Model 1 adjusted for sex.
‡Model 2 adjusted for sex, history of abnormalities in pregnancy, maternal smoking, paternal smoking, maternal history of allergy, body mass index before pregnancy, maternal age, maternal level of education, paternal level of education, family income, pet keeping, pregnancy complications, maternal drinking and parity.
